# Effect of “606” on the Eye; Seven Cases of Serious Eye Complications Following Its Use

**Published:** 1914-05

**Authors:** 


Effect of "606" on the Eye; Seven Cases of Serious Eye Complications Following Its Use.—P. S. McAdams, in summing up the effects of "606" on the eye, says that no case of injury to the healthy eye has been proved. The consensus is that it is innocuous to the healthy eye. A favorable result is to be expected from the salvarsan in syphilitic disease of the iris, the choroid, retina, and optic nerve in syphilitic paralysis of the ocular muscles, and sometimes in interstitial keratitis. It is an effective specific remedy in syphilis of the eye especially indicated when speedy aid is urgent. It is not a panacea for ocular syphilis and cannot entirely replace other preparations. No benefit is produced in simple, primary optic atrophy.

